# Exosomal miRNAs as Biomarkers for Prostate Cancer

**DOI:** 10.3389/fgene.2013.00036

**Published:** 2013-03-21

**Authors:** Nina Pettersen Hessvik, Kirsten Sandvig, Alicia Llorente

**Affiliations:** ^1^Department of Biochemistry, Institute for Cancer Research, Oslo University Hospital – The Norwegian Radium HospitalOslo, Norway; ^2^Centre for Cancer Biomedicine, Faculty of Medicine, University of OsloOslo, Norway; ^3^Department of Biosciences, University of OsloOslo, Norway

**Keywords:** exosomes, microvesicles, extracellular miRNA, prostate cancer, biofluids, biomarkers

## Abstract

miRNAs are small non-coding RNAs that finely regulate gene expression in cells. Alterations in miRNA expression have been associated with development of cancer, and miRNAs are now being investigated as biomarkers for cancer as well as other diseases. Recently, miRNAs have been found outside cells in body fluids. Extracellular miRNAs exist in different forms – associated with Ago2 proteins, loaded into extracellular vesicles (exosomes, microvesicles, or apoptotic bodies) or into high density lipoprotein particles. These extracellular miRNAs are probably products of distinct cellular processes, and might therefore play different roles. However, their functions *in vivo* are currently unknown. In spite of this, they are considered as promising, non-invasive diagnostic, and prognostic tools. Prostate cancer is the most common cancer in men in the Western world, but the currently used biomarker (prostate specific antigen) has low specificity. Therefore, novel biomarkers are highly needed. In this review we will discuss possible biological functions of extracellular miRNAs, as well as the potential use of miRNAs from extracellular vesicles as biomarkers for prostate cancer.

## Introduction

miRNAs are 19–23 nucleotides long non-coding RNAs that play important gene-regulatory roles (Carrington and Ambros, 2003; Bartel, 2004). These small RNAs downregulate gene expression through incorporation into the RNA-induced silencing complex (RISC), which then binds to partially complementary sites mainly in the 3′untranslated region (3′UTR) of their mRNA targets (Bartel, 2004). Depending on pairing complementarity, miRNAs operate through translation repression, mRNA cleavage or destabilization, or a combination of these routes (Lee et al., 1993; Wightman et al., 1993; Hutvagner and Zamore, 2002; Song et al., 2004; Lim et al., 2005). Several cellular processes like proliferation, differentiation, and apoptosis are shown to be regulated by miRNAs (Bartel, 2004) and miRNAs are found aberrantly expressed in many types of cancer (Calin et al., 2005; Iorio et al., 2005; Lu et al., 2005; Volinia et al., 2006; Porkka et al., 2007; Croce, 2009). Moreover, studies using knockout mice and transgenic mice overexpressing certain miRNAs indicate that miRNAs do contribute to cancer development (Mu et al., 2009; Hatley et al., 2010; Medina et al., 2010). It has been shown that miRNAs can act both as oncogenes and tumor suppressors, and that they participate in cancer development by regulating cell cycle, cellular senescence, DNA damage response, and apoptosis (Lima et al., 2011; Jansson and Lund, 2012).

Prostate cancer is the most commonly diagnosed cancer in men and the second leading cause of death among men with cancer in the Western world (Ferlay et al., 2007; Jemal et al., 2009). A protein mainly secreted by prostate cells, prostate specific antigen (PSA), has been used as a blood-based biomarker for prostate cancer for several decades. Even though PSA is a valuable tool, it lacks specificity and is therefore not considered an optimal biomarker (Nogueira et al., 2010). Thus, new and specific markers for prostate cancer are highly needed. Several miRNA expression profiles have been reported for prostate cancer, showing altered expression levels in prostate cancer tissue compared to control tissue (Lu et al., 2005; Volinia et al., 2006; Ozen et al., 2007; Porkka et al., 2007; Ambs et al., 2008; Szczyrba et al., 2010). Importantly, miRNAs are currently being investigated as prognostic and diagnostic tools for prostate and other types of cancer (Sørensen and Ørntoft, 2009; Kuner et al., 2013).

## Forms of Extracellular miRNAs

**Figure 1:**
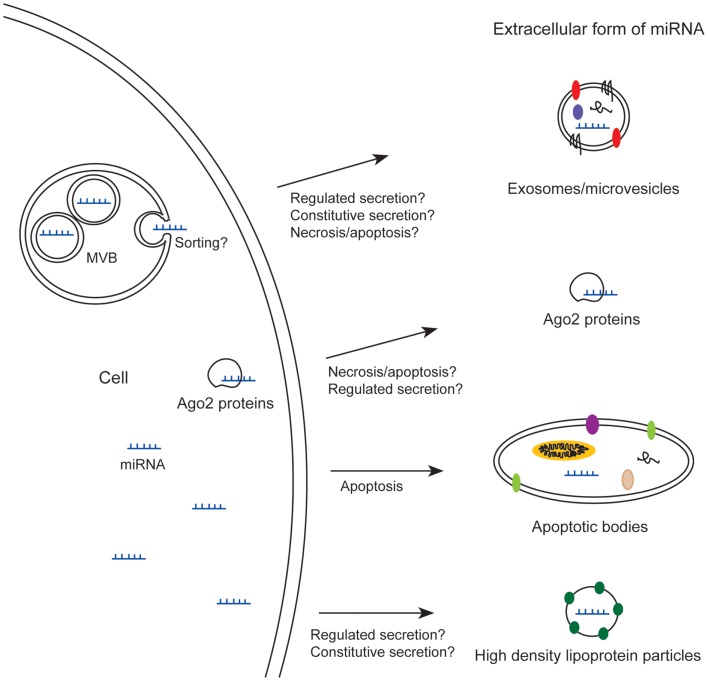
**Extracellular miRNAs exist in different forms; loaded into exosomes/microvesicles, apoptotic bodies, or high density lipoprotein particles, or associated with Ago2 proteins**. MVB, multivesicular body.

Recently, miRNAs have been identified in the medium from cultured cells (Valadi et al., 2007) and in many body fluids like blood (Chim et al., 2008; Lawrie et al., 2008), urine (Hanke et al., 2010), saliva (Park et al., 2009), breast milk (Kosaka et al., 2010b; Weber et al., 2010), and seminal plasma (Weber et al., 2010). Some of these miRNAs appear in extracellular stable forms, which render them interesting as biomarkers for cancer and other diseases. Extracellular miRNA can be found in different forms (Figure 1); some miRNAs are loaded into exosomes or microvesicles (Hunter et al., 2008; Skog et al., 2008; Lasser et al., 2011; Gallo et al., 2012; Hessvik et al., 2012), into apoptotic bodies (Zernecke et al., 2009), or into high density lipoprotein particles (Vickers et al., 2011), whereas others are associated with Ago2 proteins – proteins which are part of RISC (Arroyo et al., 2011; Turchinovich et al., 2011). Common to all these forms is that the miRNAs are not degraded by RNase treatment (Valadi et al., 2007; Turchinovich et al., 2011). First, the stability of extracellular miRNAs against RNases was thought to be mainly due to incorporation of miRNAs into small extracellular vesicles (Valadi et al., 2007; Hunter et al., 2008). However, some reports have shown that most of the extracellular miRNAs in plasma and cell culture media are found outside vesicles, in stable complexes with Ago2 proteins (Arroyo et al., 2011; Turchinovich et al., 2011), although a recent publication showed that the majority of miRNAs in serum and saliva is enclosed in exosomes (Gallo et al., 2012).

## Extracellular Vesicles

As mentioned, miRNAs have been found in several types of extracellular vesicles. Extracellular vesicles are mainly classified based on the different mechanism of release, but a consensus in the terminology used to name these small vesicles is still lacking. Normally, exosomes are defined as small membrane vesicles with a diameter of 40–100 nm that are secreted when multivesicular bodies (MVBs) fuse with the plasma membrane (Pan et al., 1985; Johnstone et al., 1987; Bobrie et al., 2011). Shedding vesicles or microparticles are 0.1–1 μm in diameter and formed by budding from the plasma membrane (Cocucci et al., 2009). To collectively describe both exosomes and shedding vesicles the term microvesicles is often used (Cocucci et al., 2009), though this term is sometimes also used to indicate either one of these types of vesicles. A third type of extracellular vesicles is apoptotic bodies, which are 1–4 μm in diameter and formed during apoptosis (Hristov et al., 2004; Zernecke et al., 2009). Recently, the term extracellular vesicles has been used to describe these three classes of vesicles as a group (Kalra et al., 2012).

## Potential Roles of Extracellular miRNAs

Whether the extracellular forms of miRNAs are simply waste products from cells or have a biological function, such as participating in intercellular communication is not yet clear. There are reports showing increased level of miRNAs in blood upon organ toxicity (Laterza et al., 2009; Zhang et al., 2010; Pritchard et al., 2012), and this could of course represent waste products. Nevertheless, since the various forms of extracellular miRNAs are probably products of distinct cellular processes, they might play different roles, and therefore it is important to distinguish between them. Apoptotic bodies are by definition formed during apoptosis. miRNA bound to Ago2 may be released from cells upon apoptosis or necrosis (Turchinovich et al., 2011), but it is not known if miRNA-Ago2 complexes also can be transported out of viable cells. This means that miRNAs bound to Ago2 proteins and miRNAs incorporated into apoptotic bodies might solely be by-products from dying cells or represent a way for dying cells to communicate with neighboring cells. They could represent a signal warning the organism about cellular dysfunction.

Shedding vesicles and exosomes are thought to be released by viable cells, though it is not ruled out whether these vesicles also are released by dying cells. Therefore, these vesicles have to a greater extent been suggested to play a role in intercellular signaling (Valadi et al., 2007; Hunter et al., 2008). Indeed, it has been shown that miRNAs can be transferred by exosomes from one cell to another *in vitro* and result in downregulation of target genes in the recipient cell (Kosaka et al., 2010a; Kogure et al., 2011; Mittelbrunn et al., 2011; Montecalvo et al., 2012). This finding is intriguing and indicates a role in intercellular communication which could have a huge impact. Yet this remains to be shown *in vivo*. Interestingly, it has been reported that injection of exosomes loaded with siRNA into mice can result in specific gene knockdown in certain cells (Alvarez Erviti et al., 2011). It has been questioned whether the concentration of exosomes in biological fluids is high enough to play a role in intercellular communication, but this does not exclude a role in autocrine or paracrine signaling (Turchinovich et al., 2011; Sverdlov, 2012). Exosomes probably exert their effect on neighboring cells, and thereby participate in creating a specific microenvironment. In this scenario, the exosomes found in body fluids would only be residual amounts, representing a secondary effect.

In addition to their conventional role in post-transcriptional gene regulation, a new role for miRNAs as signaling molecules has recently been described by two independent groups. Interestingly, extracellular let-7 was shown to activate Toll-like receptor 7 in neurons and induce neurodegeneration (Lehmann et al., 2012). By another group, exosomal miR-21 and miR-29a was shown to activate Toll-like receptor 7 and 8 in immune cells, triggering a prometastatic inflammatory response that may lead to tumor growth and metastasis (Fabbri et al., 2012). Thus, extracellular miRNAs could be important regulators of tumor microenvironment as well as exacerbate CNS damage, through agonistic effect on Toll-like receptor 7 and 8.

Another possible role for miRNAs in exosomes and MVBs is that they might function together with the RNAi machinery. RISC proteins have been shown to be associated with MVBs and exosomes (Gibbings et al., 2009; Lee et al., 2009). Moreover, blocking MVB formation by depletion of ESCRT (endosomal sorting complex required for transport) components has been reported to result in impaired miRNA silencing, indicating a role in RNAi dynamics (Gibbings et al., 2009; Lee et al., 2009).

## Are miRNAs Sorted into Exosomes?

Another debated issue in the field is whether miRNAs are sorted into MVBs and exosomes or not. Several reports have shown that certain miRNAs are selectively identified or expressed at a higher level in exosomes than in parent cells (Valadi et al., 2007; Ohshima et al., 2010; Kogure et al., 2011; Mittelbrunn et al., 2011; Hessvik et al., 2012), indicating a sorting of miRNAs into MVBs. The opposite situation has also been reported; for example one study showed that only 2% of the most abundant miRNA in a breast cancer cell line, miR-720, was found extracellularly, whereas many other miRNAs were presented at comparable levels in the cellular and extracellular populations (Pigati et al., 2010). The findings that certain miRNAs are either enriched in exosomes or retained in cells, indicate that exosomal miRNAs are not simply unsorted waste products from cells. The mechanisms controlling the selection of miRNAs into exosomes or the retention of miRNAs inside cells still remain unknown. Though, it cannot be excluded that the higher expression level of certain miRNAs in exosomes is due to shielding of miRNAs from degradation by exosome membranes, and not due to a sorting mechanism. Moreover, Kim et al. (2012) recently reported that small RNAs with low GC content can be lost during RNA isolation from samples with low RNA content, indicating methodological challenges when working with extracellular miRNAs.

Higher levels of exosomes are found in plasma from cancer patients compared to control individuals (Rabinowits et al., 2009; Tavoosidana et al., 2011), suggesting that cancer cells secrete more exosomes than non-cancerous cells. Therefore, measuring exosomal miRNAs could result in less background from normal cells, and they might then serve as superior biomarkers compared to other extracellular miRNAs. However, the currently used protocols for exosome isolation are extensive and time-consuming. New and faster isolation methods need to be established before exosomal miRNAs can be used in routine diagnostics.

## miRNAs and Prostate Cancer

The first profiling of miRNAs in prostate cancer was published in 2007 (Porkka et al., 2007). In this study prostate cancer cell lines and prostate tissue from both benign prostatic hyperplasia and prostate cancer patients were examined. Today, the miRNA expression in prostate cancer has been published in more than 100 reports, showing promising results for miRNAs as tissue-based biomarkers for this disease (Catto et al., 2011). Measurement of extracellular miRNAs obtained from biological fluids constitutes a non-invasive approach for cancer detection and may therefore be preferable. Indeed, several studies (Table 1, and discussed below) have investigated the miRNA profile in serum/plasma from prostate cancer patients, pointing toward the use of miRNAs as blood-based biomarkers (Mitchell et al., 2008; Lodes et al., 2009; Brase et al., 2011; Moltzahn et al., 2011).

**Table 1:** **Studies of extracellular miRNAs in body fluids from prostate cancer patients**.

Promising miRNA	Deregulation	Body fluid	Study design	Number of participants	Reference
let-7a	↑	Whole blood	PCa vs. healthy controls	83	Heneghan et al. (2010)
let-7c	↓	Plasma	PCa vs. BPH and healthy controls	Screening: 42	Chen et al. (2012)
				Validation: 178	
let-7e	↓	Plasma	PCa vs. BPH and healthy controls	Screening: 42	Chen et al. (2012)
				Validation: 178	
let-7i	↑	Serum	Localized PCa vs. BPH and healthy controls	83	Mahn et al. (2011)
miR-16	↑	Serum	Stage 3 and 4 PCa patients vs. healthy controls	13	Lodes et al. (2009)
miR-20a	↑	Plasma	PCa: high risk vs. intermediate risk vs. low risk	82	Shen et al. (2012)
miR-21	↑	Plasma	PCa: high risk vs. intermediate risk vs. low risk	82	Shen et al. (2012)
	↑	Plasma	Metastatic PCa vs. localized/local advanced PCa vs. healthy controls	71	Yaman Agaoglu et al. (2011)
	↑	Serum	Hormone-refractory PCa vs. androgen dependent PCa vs. localized PCa vs. BPH	56	Zhang et al. (2011)
miR-24	↓	Serum	PCa vs. healthy controls	48	Moltzahn et al. (2011)
miR-26a	↑	Serum	Localized PCa vs. BPH and healthy controls	83	Mahn et al. (2011)
miR-26b	↓	Serum	PCa vs. healthy controls	48	Moltzahn et al. (2011)
miR-30c	↓	Plasma	PCa vs. BPH and healthy controls	Screening: 42	Chen et al. (2012)
				Validation: 178	
	↓	Serum	PCa vs. healthy controls	48	Moltzahn et al. (2011)
miR-34b	↑	Serum	Stage 3 and 4 PCa patients vs. healthy controls	13	Lodes et al. (2009)
miR-92a	↑	Serum	Stage 3 and 4 PCa patients vs. healthy controls	13	Lodes et al. (2009)
miR-92b	↑	Serum	Stage 3 and 4 PCa patients vs. healthy controls	13	Lodes et al. (2009)
miR-93	↑	Serum	PCa vs. healthy controls	48	Moltzahn et al. (2011)
miR-103	↑	Serum	Stage 3 and 4 PCa patients vs. healthy controls	13	Lodes et al. (2009)
miR-106a	↑	Serum	PCa vs. healthy controls	48	Moltzahn et al. (2011)
miR-107	↑	Plasma (microvesicles)	PCa vs. healthy controls	Screening: 106	Bryant et al. (2012)
	↑	Urine (cells)	PCa vs. healthy controls	135	Bryant et al. (2012)
	↑	Serum	Stage 3 and 4 PCa patients vs. healthy controls	13	Lodes et al. (2009)
miR-130b	↑	Plasma (microvesicles)	PCa vs. healthy controls	Screening: 106	Bryant et al. (2012)
miR-141	↑	Serum	Metastatic PCa vs. localized PCa	Screening: 21	Brase et al. (2011)
				Validation: 116	
	↑	Plasma (microvesicles)	PCa vs. healthy controls	Screening: 106	Bryant et al. (2012)
	↑	Serum (microvesicles)	Metastatic vs. localized PCa	Validation: 119	Bryant et al. (2012)
	↑	Serum	Metastatic PCa vs. healthy controls	50	Mitchell et al. (2008)
	↑	Serum	Metastatic PCa vs. healthy controls	50	Selth et al. (2012)
	↑	Plasma	Metastatic PCa vs. localized/local advanced PCa vs. healthy controls	71	Yaman Agaoglu et al. (2011)
miR-145	↓	Whole blood	PCa vs. healthy controls	83	Heneghan et al. (2010)
	↑	Plasma	PCa: high risk vs. intermediate risk vs. low risk	82	Shen et al. (2012)
miR-155	↓	Whole blood	PCa vs. healthy controls	83	Heneghan et al. (2010)
miR-181a-2*	↓	Plasma (microvesicles)	PCa vs. healthy controls	Screening: 106	Bryant et al. (2012)
miR-195	↑	Serum	Localized PCa vs. BPH and healthy controls	83	Mahn et al. (2011)
miR-197	↑	Serum	Stage 3 and 4 PCa patients vs. healthy controls	13	Lodes et al. (2009)
miR-221	↑	Plasma	PCa: high risk vs. intermediate risk vs. low risk	82	Shen et al. (2012)
	↑	Plasma	Metastatic PCa vs. localized/local advanced PCa vs. healthy controls	71	Yaman Agaoglu et al. (2011)
	↑	Plasma	PCa vs. healthy controls	43	Zheng et al. (2012)
miR-223	↓	Serum	PCa vs. healthy controls	48	Moltzahn et al. (2011)
miR-298	↑	Serum	Metastatic PCa vs. healthy controls	50	Selth et al. (2012)
miR-301a	↑	Plasma (microvesicles)	PCa vs. healthy controls	Screening: 106	Bryant et al. (2012)
miR-326	↑	Plasma (microvesicles)	PCa vs. healthy controls	Screening: 106	Bryant et al. (2012)
miR-328	↑	Serum	Stage 3 and 4 PCa patients vs. healthy controls	13	Lodes et al. (2009)
miR-331-3p	↑	Plasma (microvesicles)	PCa vs. healthy controls	Screening: 106	Bryant et al. (2012)
miR-346	↑	Serum	Metastatic PCa vs. healthy controls	50	Selth et al. (2012)
miR-375	↑	Serum	Metastatic PCa vs. localized PCa	Screening: 21	Brase et al. (2011)
				Validation: 116	
	↑	Serum (microvesicles)	Metastatic vs. localized PCa	Validation: 119	Bryant et al. (2012)
	↑	Serum	Metastatic PCa vs. healthy controls	50	Selth et al. (2012)
miR-432	↑	Plasma (microvesicles)	PCa vs. healthy controls	Screening: 106	Bryant et al. (2012)
miR-485-3p	↑	Serum	Stage 3 and 4 PCa patients vs. healthy controls	13	Lodes et al. (2009)
miR-486-5p	↑	Serum	Stage 3 and 4 PCa patients vs. healthy controls	13	Lodes et al. (2009)
miR-574-3p	↑	Plasma (microvesicles)	PCa vs. healthy controls	Screening: 106	Bryant et al. (2012)
	↑	Urine (cells)	PCa vs. healthy controls	135	Bryant et al. (2012)
	↑	Serum	Stage 3 and 4 PCa patients vs. healthy controls	13	Lodes et al. (2009)
miR-622	↑	Plasma	PCa vs. BPH and healthy controls	Screening: 42	Chen et al. (2012)
				Validation: 178	
miR-625*	↑	Plasma (microvesicles)	PCa vs. healthy controls	Screening: 106	Bryant et al. (2012)
miR-636	↑	Serum	Stage 3 and 4 PCa patients vs. healthy controls	13	Lodes et al. (2009)
miR-640	↑	Serum	Stage 3 and 4 PCa patients vs. healthy controls	13	Lodes et al. (2009)
miR-766	↑	Serum	Stage 3 and 4 PCa patients vs. healthy controls	13	Lodes et al. (2009)
miR-874	↑	Serum	PCa vs. healthy controls	48	Moltzahn et al. (2011)
miR-885-5p	↑	Serum	Stage 3 and 4 PCa patients vs. healthy controls	13	Lodes et al. (2009)
miR-1207-5p	↑	Serum	PCa vs. healthy controls	48	Moltzahn et al. (2011)
miR-1274a	↑	Serum	PCa vs. healthy controls	48	Moltzahn et al. (2011)
miR-1285	↑	Plasma	PCa vs. BPH and healthy controls	Screening: 42	Chen et al. (2012)
				Validation: 178	
miR-2110	↑	Plasma (microvesicles)	PCa vs. healthy controls	Screening: 106	Bryant et al. (2012)

*miRNAs ordered by increasing name number. PCa, prostate cancer; BPH, benign prostatic hyperplasia. Asterisks are part of the miRNA names*.

In the first study, the level of six miRNAs in serum samples from 25 patients with metastatic prostate cancer and 25 healthy controls was analyzed. The authors found that miR-141 was overexpressed in the prostate cancer group compared to the control group, and that this miRNA had the greatest differential expression among the six tested miRNAs (Mitchell et al., 2008). Later, 667 miRNAs in serum samples from patients with metastatic (*n* = 7) or localized prostate cancer (*n* = 14) were screened. In this study 69 miRNAs were found to be higher expressed in the metastatic tumor group compared to the primary cancer group. Five of the upregulated miRNAs (miR-375, miR-9*, miR-141, miR-200b, and miR-516a-3p) were further validated, resulting in the identification of miR-375 and miR-141 as the best markers for high risk prostate cancer (Brase et al., 2011). Another study analyzed the expression of miR-21, miR-141, and miR-221 in plasma from patients with localized/local advanced (*n* = 26) or metastatic (*n* = 25) prostate cancer and healthy controls (*n* = 20). A higher expression level of miR-21 and miR-221, but not statistically significant for miR-141, was observed in plasma from prostate cancer patients compared to controls. Still, all three miRNAs were significantly higher in patients with metastatic prostate cancer than in patients with localized/local advanced disease (Yaman Agaoglu et al., 2011). In a study by Lodes et al. 547 miRNAs were screened, 15 miRNAs (miR-16, -92a, -103, -107, -197, -34b, -328, -485-3p, -486-5p, -92b, -574-3p, -636, -640, -766, -885-5p) were found to be overexpressed in serum from stage 3 and 4 prostate cancer patients (*n* = 5) compared to eight healthy controls. They also showed a slightly elevated level of miR-141 in stage 3 and 4 prostate cancer patient serum (Lodes et al., 2009). Selth et al. analyzed the expression of 10 miRNAs in serum from patients with metastatic castration-resistant prostate cancer (*n* = 25) and healthy controls (*n* = 25). They found that miR-141, miR-298, miR-346, and miR-375 were upregulated in serum from prostate patients compared with controls (Selth et al., 2012). Though these independent studies point toward plasma/serum-derived miR-141 and miR-375 as biomarkers, Mahn et al. (2011) did not succeed in detecting miR-141 in serum samples.

In the study by Mahn et al. the expression of four miRNAs in serum from 37 patients with localized prostate cancer, eight with metastatic prostate cancer, 18 with benign prostatic hyperplasia, and 20 healthy controls was analyzed. They found miR-26a, miR-195, and let-7i to be upregulated in patients with prostate cancer compared with patients with benign prostatic hyperplasia. Moreover, they also showed that miR-26a levels could discriminate patients with prostate cancer from patients with benign prostatic hyperplasia (Mahn et al., 2011). Another report showed results from screening of 384 miRNAs in serum from 36 prostate cancer patients and 12 healthy controls. Five miRNAs (miR-874, -1274a, -1207-5p, -93, and -106a) were identified to be upregulated and four (miR-223, -26b, -30c, and -24) downregulated in cancer patients compared to controls (Moltzahn et al., 2011). Heneghan et al. (2010) analyzed the expression of seven miRNAs and found a decreased level of miR-145 and miR-155 and an increased level of let-7a in whole blood from prostate cancer patients (*n* = 20) compared to healthy controls (*n* = 63).

Although a higher level of miR-21 in plasma from prostate cancer patients compared to controls has been observed (Yaman Agaoglu et al., 2011), Zhang et al. did not find a significant difference in the level of serum-derived miR-21 between patients with benign prostatic hyperplasia (*n* = 6), localized (*n* = 20), and androgen dependent prostate cancer (*n* = 20). Still, higher expression of miR-21 was detected in patients with hormone-refractory prostate cancer (*n* = 10), especially in patients resistant to docetaxel-based chemotherapy, suggesting a potential role for miRNAs as markers for disease progression and response to treatment (Zhang et al., 2011). In another study, the level of miR-221 in plasma samples from healthy controls (*n* = 20), androgen dependent (*n* = 15), and androgen independent (*n* = 8) prostate cancer patients was analyzed. In accordance with the findings by Yaman Agaoglu et al. the authors observed increased expression of miR-221 in plasma samples from prostate cancer patients compared with healthy controls, whereas the level of miR-221 was increased in androgen dependent compared to androgen independent prostate cancer patients (Zheng et al., 2012).

Chen et al. screened the miRNA expression in plasma from 25 prostate cancer patients and 17 patients with benign prostatic hyperplasia and validated candidate miRNAs in a larger independent cohort (80 prostate cancer patients, 44 patients with benign prostatic hyperplasia, and 54 healthy controls). These authors showed that five miRNAs (let-7c, let-7e, miR-30c, miR-622, and miR-1285) could differentiate patients with prostate cancer from patients with benign prostatic hyperplasia and healthy controls. They suggested that a panel of the five described miRNAs could distinguish these patient groups from each other with higher sensitivity and specificity compared to one single miRNA (Chen et al., 2012). Another group also suggested a combination of several miRNAs as biomarker after analyzing miRNA levels in plasma samples from 82 prostate cancer patients with varied aggressiveness. In this report the combination of miR-20a, miR-21, miR-145, and miR-221 was shown to distinguish prostate cancer patients with high risk of aggressiveness from those with low risk (Shen et al., 2012).

Neither of the mentioned studies describes in which form the extracellular miRNAs were packaged. In only one study miRNAs from plasma- and serum-derived microvesicles were analyzed, though the vesicles were not well characterized. Eleven miRNAs were found to be differently expressed in prostate cancer patients compared to healthy controls (miR-107, -130b, -141, -181a-2*, -2110, -301a, -326, -331-3p, -432, -574-3p, and -625*), and 16 miRNAs were upregulated in patients with metastases compared to patients without metastases. The association of miR-141 and miR-375 with metastatic prostate cancer was also confirmed. The authors selected five miRNAs that were analyzed in urine samples, showing that miR-107 and miR-574-3p were found in higher concentrations in the urine of men with prostate cancer compared with healthy controls. However, these miRNAs were not really extracellular, since cell pellets from urine were used in this part of the study (Bryant et al., 2012). Recently, we described the miRNA profile in exosomes from the PC-3 metastatic prostate cancer cell line. Among the aforementioned miRNAs suggested as biomarker candidates in clinical studies, we identified 36 in exosomes from PC-3 cells (miR-141, -9*, -200b, -21, -221, -16, -92a, -103, -107, -197, -92b, -574-3p, -885-5p, -298, -26a, -1274a, -106a, -26b, -30c, -24, let-7i, let-7a, let-7c, let-7e, miR-1285, -20a, -107, -130b, -301a, -331-3p, -625, -485-3p, -874, -155, -181a-2*, and -326) (Hessvik et al., 2012).

As described, an emerging amount of evidence points toward a potential use of blood-based miRNAs as diagnostic and prognostic biomarkers for prostate cancer (Table 1). Nevertheless, the data from the clinical studies are to some extent inconsistent. This may not be surprising due to the fact that these studies differ with respect to experimental design and patient cohort. Most of these studies are small-scale, with varying degree of patient description and few are focusing on cancer-related death or progression free survival as endpoints. At least one study included patients under treatment with chemotherapy, therefore it is uncertain whether the altered miRNA levels were due to the chemotherapy or due to the tumor itself (Lodes et al., 2009). In addition, different platforms have been used to quantify miRNA levels, which also could explain some of the conflicting data. High throughput platforms always have biases and confirmation with a secondary analytical approach, such as RT-qPCR, should be performed to validate the results. Isolation of extracellular miRNAs using different methods can contribute to variation due to low recovery of certain miRNA species, and quantification of extracellular miRNAs are challenging due to lack of standards for quality control, normalization, and statistical analysis. Together, these factors might explain most of the conflicting results.

Blood is the body fluid that traditionally has been used as the main source of cancer biomarkers, though the use of urine in cancer diagnosis and prognosis is growing. Choosing urine as a source of biomarkers has several advantages compared to blood; it is non-invasive, easily obtained in large quantities, and the composition of urine is less complex. Particularly important for prostate cancer is the fact that the composition of urine reflects alterations in the urogenital system. However, dilution of the miRNAs could be a possible drawback with the use of urine as a source of biomarkers. To our knowledge there are currently no publications describing the miRNA content of urinary exosomes in prostate cancer.

## Conclusion

Intensive research is put into the hunt for new diagnostic and prognostic tools for prostate cancer. Representing a non-invasive approach, measurement of extracellular miRNAs in blood, urine, or other biological fluids might turn out as a valuable strategy. Studies on blood-based miRNAs as biomarkers for prostate cancer are emerging, but most of these are small-scale, vary in methodology, and lack a characterization of the form in which the extracellular miRNAs are found. Standards for miRNA quantification need to be established and larger validation studies need to be performed before conclusions can be drawn on the diagnostic potential of specific miRNAs. It might be important to distinguish between different forms of extracellular miRNAs, both for diagnosis and for understanding possible biological functions. More and large-scale studies on exosomal miRNAs in biological fluids from prostate cancer patients and healthy controls are therefore needed. Although there is still very limited knowledge about the biological roles of extracellular miRNAs and extracellular vesicles, they are currently emerging as an important source of biomarkers.

## Conflict of Interest Statement

The authors declare that the research was conducted in the absence of any commercial or financial relationships that could be construed as a potential conflict of interest.
